# Distinct dedifferentiation processes affect caveolin-1 expression in hepatocytes

**DOI:** 10.1186/1478-811X-11-6

**Published:** 2013-01-22

**Authors:** Christoph Meyer, Johanna Dzieran, Yan Liu, Felizitas Schindler, Stefan Munker, Alexandra Müller, Cédric Coulouarn, Steven Dooley

**Affiliations:** 1 Department of Medicine II, Section Molecular Hepatology, Medical Faculty Mannheim, Heidelberg University, Mannheim, Germany; 2 Department of Molecular Cell Biology and Centre for Biomedical Genetics, Leiden University Medical Center, 2300 RC, Leiden, The Netherlands; 3 INSERM, UMR991, University of Rennes 1, Pontchaillou University Hospital, F-35033, 2 rue Henri Le Guilloux, Rennes, France

## Abstract

**Background:**

Dedifferentiation and loss of hepatocyte polarity during primary culture of hepatocytes are major drawbacks for metabolic analyses. As a prominent profibrotic cytokine and potent inducer of epithelial mesenchymal transition (EMT), TGF-β contributes to these processes in liver epithelial cells. Yet, a distinction between culture dependent and TGF-β driven hepatocyte dedifferentiation has not been shown to date.

**Results:**

Here, we show that in both settings, mesenchymal markers are induced. However, upregulation of Snai1 and downregulation of E-Cadherin are restricted to TGF-β effects, neglecting a full EMT of culture dependent hepatocyte dedifferentiation. Mechanistically, the latter is mediated via FAK/Src/ERK/AKT pathways leading to the induction of the oncogene caveolin-1 (Cav1). Cav1 was recently proposed as a new EMT marker, but our results demonstrate Cav1 is not up-regulated in TGF-β mediated hepatocyte EMT, thus limiting validity of its use for this purpose. Importantly, marking differences on Cav1 expression exist in HCC cell lines. Whereas well differentiated HCC cell lines exhibit low and inducible Cav1 protein levels - by TGF-β in a FAK/Src dependent manner, poorly differentiated cell lines display high Cav1 expression levels which are not further modulated by TGF-β.

**Conclusions:**

This study draws a detailed distinction between intrinsic and TGF-β mediated hepatocyte dedifferentiation and elucidates cellular pathways involved. Additionally, by evaluating the regulation of the oncogene Cav1, we provide evidence to argue against Cav1 as a reliable EMT marker.

## Background

Primary cultures of human or rodent hepatocytes are of particular value for investigating drug metabolism and toxicity. However, basic functional hepatocyte features such as bile canaliculi formation, bile secretion, polarity and metabolic activities (including detoxification by Cyp and other drug-metabolizing enzymes) are rapidly lost during culture on a collagen layer (monolayer or 2D culture). To overcome these limitations, alternative hepatocyte culture systems have been developed, including co-culture systems, bioreactors and 3D systems, where hepatocytes are embedded in a soft collagen matrix [1,2].

However, hepatocyte culture on a single stiff collagen surface possesses interesting features for researchers. Indeed, monolayer culture of primary hepatocytes offers an astonishing view on cell plasticity, and allows delineation of pathways regulating hepatocyte polarity and homeostasis [3]. Even though hepatocyte dedifferentiation in culture has not been deeply investigated with respect to epithelial to mesenchymal transition (EMT) so far, the switch of cell morphology toward a fibroblastoid phenotype and the induction of EMT-associated collagen I expression argues for such process *in vitro*[4].

Transforming growth factor beta (TGF-β), a pleiotropic cytokine, is considered as the most potent profibrotic mediator in diverse organ diseases [5]. It is also recognized as a strong EMT inducer in various epithelial cell types including hepatocytes cultured on a stiff collagen matrix [3,6,7]. These observations raise the question whether TGF-β acts as an accelerator of the intrinsic dedifferentiation process or whether culture and TGF-β mediated EMT are distinct processes.

Caveolin-1 is the forming protein of caveolae, invaginations of the plasma membrane, and defines a particular endocytic route [8]. Although caveolin-1 expression is elevated in hepatocellular carcinoma (HCC), its function as a tumor suppressor or promoter is still debated [9-12]. Recently, caveolin-1 was shown as being required for mitochondrial function and lack of caveolin-1 resulted in apoptotic susceptibility [13]. Thus, an increase of caveolin-1 might serve for enhanced mitochondrial stability under stressful conditions like liver fibrosis and inflammation as well as for an additional survival strategy for cancer cells.

TGF-β was shown to enhance tumor growth under certain conditions and has recently been demonstrated to increase caveolin-1 expression in the pluripotent human embryonic carcinoma cell line NT2/D1 as well as in murine mammary epithelial cells (NMuMG) [14].

In this study, we define pathways responsible for caveolin-1 expression in dedifferentiating hepatocytes and discern important differences of intrinsic and TGF-β induced hepatocyte dedifferentiation. Whereas TGF-β driven hepatocyte EMT is not accompanied by increased caveolin-1 levels, intrinsic dedifferentiation is mediated via FAK/Src and the downstream pathways ERK1/2 and AKT, causing a tremendous increase of caveolin-1 expression. Further, the latter process is completely independent of the Snai1 transcription factor, a major EMT mediator. Finally, in contrast to primary hepatocytes, TGF-β is capable of inducing caveolin-1 expression in well differentiated, but not in dedifferentiated HCC cell lines.

## Results

### Culture induced hepatocyte dedifferentiation leads to upregulation of mesenchymal markers and caveolin-1

**Figure 1:**
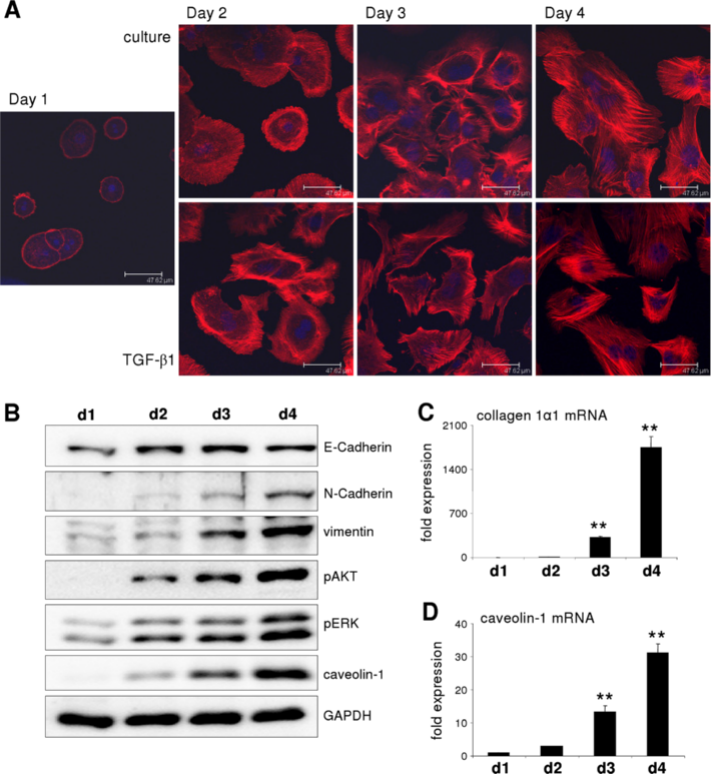
**Intrinsic hepatocyte dedifferentiation is accompanied with upregulation of mesenchymal markers.** (**A**) Time course of cultured hepatocytes stained with phalloidin. Starting on day 2, hepatocytes loose polar structure. Lower panel shows cells treated with TGF-β1 leading to similar actin remodeling and reduced cell-cell contacts over time. (**B**) Western blot showing a time course of murine hepatocytes cultured in monolayer on a stiff collagen matrix. Mesenchymal markers are induced and AKT and ERK signaling pathways are activated over time. Caveolin-1 is strongly upregulated during culture. (**C**) Collagen 1α1 (p < 0.001 at d3 and d4) and (**D**) caveolin-1 mRNA (p < 0.001 at d3 and d4) are upregulated in culture over time.

Hepatocytes cultured on collagen monolayer rapidly dedifferentiate. This was nicely illustrated by staining of F-actin fibres utilizing phalloidin. At day 1, primary hepatocytes still exhibited a polar organization with actin fibers forming a belt on the inner side of the cell membrane (Figure 1A). Starting at day 2, hepatocytes spread on the substratum and re-structure the actin cytoskeleton. TGF-β treatment enhanced cytoskeleton remodelling in hepatocytes which finally culminated in fibroblastoid morphology with limited cell-cell contacts (resembling EMT). In contrast, although culture induced dedifferentiation affected the actin structures, cell-cell contacts were still visible (Figure 1A).

Besides the loss of polar shape during culture, mesenchymal markers were expressed, as illustrated by the induction of N-Cadherin and Vimentin within four days of culture. Furthermore, this process correlated with the activation of MAPK/ERK and AKT signaling pathways (Figure 1B). A striking increase in Collagen 1α1 mRNA expression level was observed after 4 days of culture (p < 0.001), thus further supporting the gain of mesenchymal properties of cultured hepatocytes (Figure 1C). Besides expression of these markers, caveolin-1 was strongly induced both at mRNA (p < 0.001 at day 3 and 4) and protein levels (Figure 1B, D). Intriguingly, protein levels of the epithelial marker E-Cadherin were not decreased during intrinsic dedifferentiation. Nevertheless, localization of E-Cadherin was affected as during dedifferentiation immunostaining demonstrated a reduced localization at cell-cell contacts/tight junctions (data not shown &[3]).

### The FAK/Src signaling pathway mediates caveolin-1 expression

**Figure 2:**
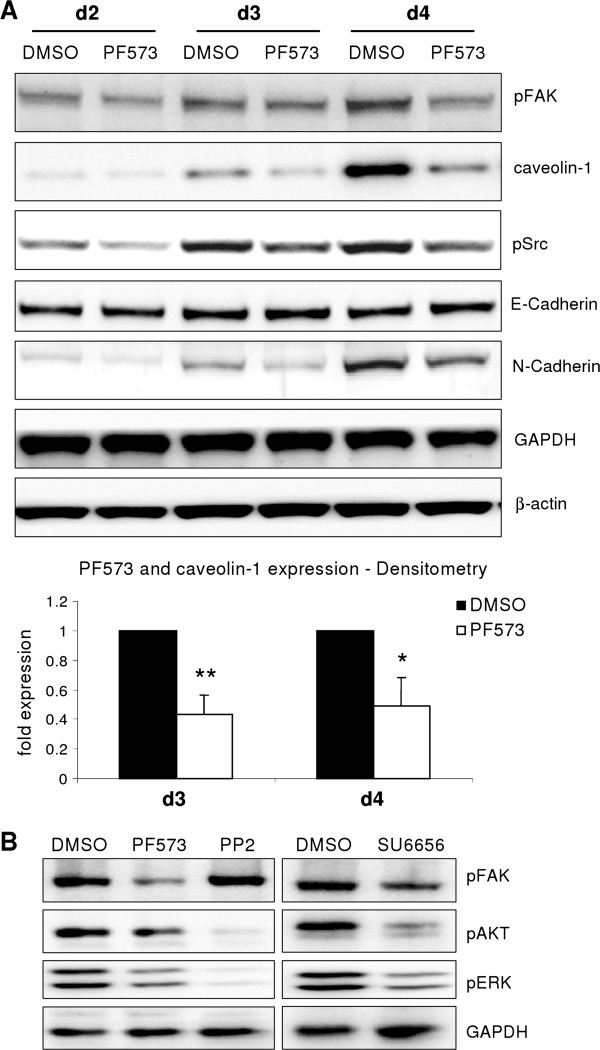
**FAK activation influences caveolin-1 induction.** (**A**) FAK and Src phosphorylation increases over time in culture. Blocking FAK phosphorylation with PF573228 (4 μM) culminates in reduced phospho-Src and N-Cadherin expression as well as attenuated caveolin-1 upregulation. E-Cadherin expression is unchanged. Caveolin-1 expression was densitometrically analyzed. A significant downregulation was observed at day 3 (p = 0.002) and day 4 (p = 0.011) (**B**) Blocking FAK (PF573228, 4 μM) leads to reduced AKT and ERK phosphorylation. Inhibition of Src kinases results in attenuated AKT and ERK phosphorylation. SU6656 (15 μM) additionally affects FAK phosphorylation, whereas PP2 (10 μM) does not. These data indicate that FAK signals via Src/ERK/AKT.

We have shown previously that the integrin/FAK/Src axis triggered hepatocyte dedifferentiation [3]. Thus, we next investigated whether this pathway also contributed to caveolin-1 induction. Inhibition of FAK was achieved using the small chemical inhibitor PF573228 which led to abrogation of FAK tyrosine 397 and to a reduction of Src tyrosine 527 phosphorylation. The blockage of FAK subsequently attenuated caveolin-1 upregulation (Figure 2A). Additionally, downstream signaling of FAK, in terms of pSrc, pAKT and pERK was affected (Figure 2B). To further elucidate that Src family members are essential in mediating activation of AKT and ERK, two Src inhibitors were applied and downstream signaling was evaluated. Inhibition of Src with SU6656 and PP2 (Figure 2B) yielded in reduced activity of ERK and AKT pathways. Application of different inhibitors of Src family members is adequate in our experimental setting due to their variant substrate specificity [15] and thus distinct downstream effects.

**Figure 3:**
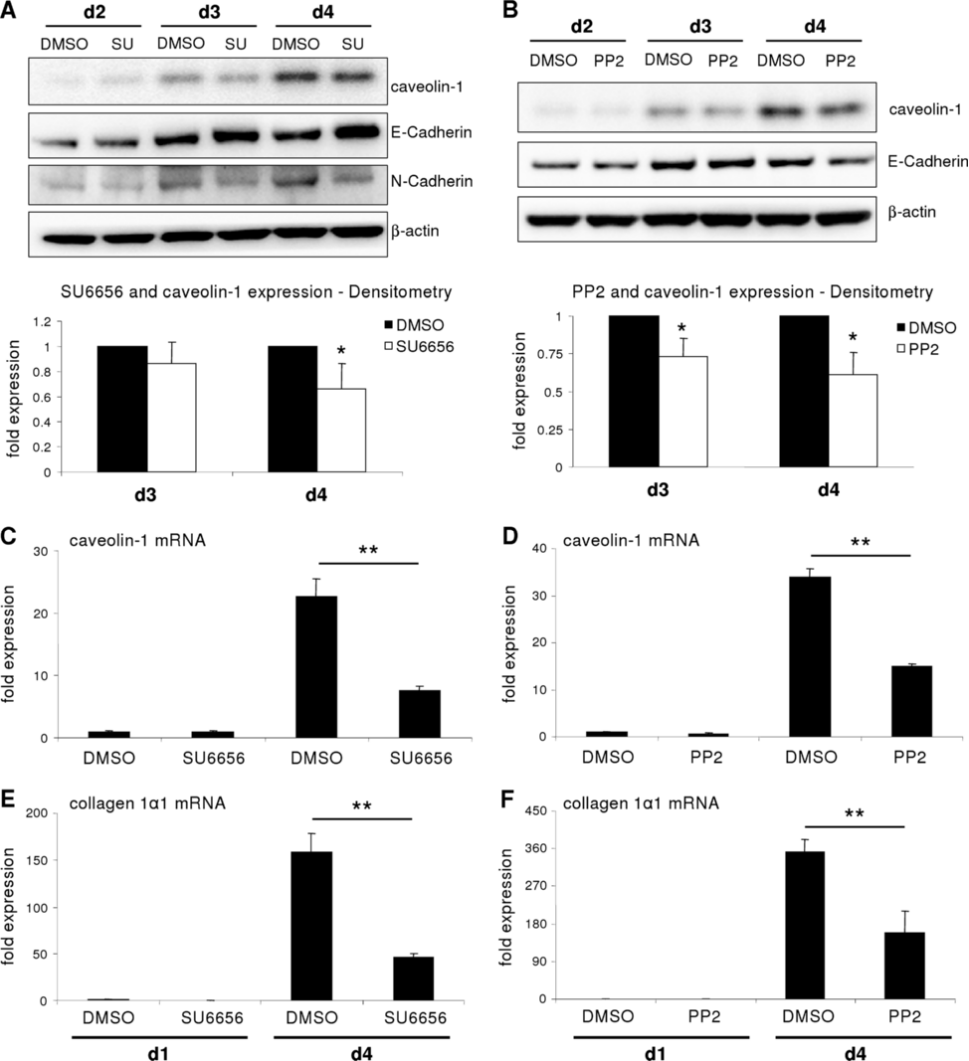
**Src is a critical mediator for caveolin-1 induction.** (**A**) SU6656 (SU; 15 μM) was used to block Src activity. Blockage yielded in reduced caveolin-1 protein (evaluated with densitometry) similar to (**B**) where PP2 (10 μM) was used. (**C**, **D**) Caveolin-1 mRNA expression is also reduced during culture in presence of inhibitors (p = 0.002 for SU6656 at d4; p < 0.001 for PP2 at d4). (**E**, **F**) Induction of Collagen 1α1 was reduced upon Src inhibition with both inhibitors (SU6656: p < 0.001; PP2: p = 0.005).

Similarly, as observed with the FAK inhibitor, Src blockage significantly prevented upregulation of caveolin-1 in hepatocytes on protein (Figure 3A, B) and mRNA levels (Figure 3C, D). Attenuated hepatocyte dedifferentiation was demonstrated by reduced expression of N-Cadherin and Collagen 1α1 mRNA, as well as sustained E-Cadherin expression when cells were cultured in presence of Src family inhibitors (Figure 3A, B, E, F).

### AKT and ERK contribute to caveolin-1 upregulation

**Figure 4:**
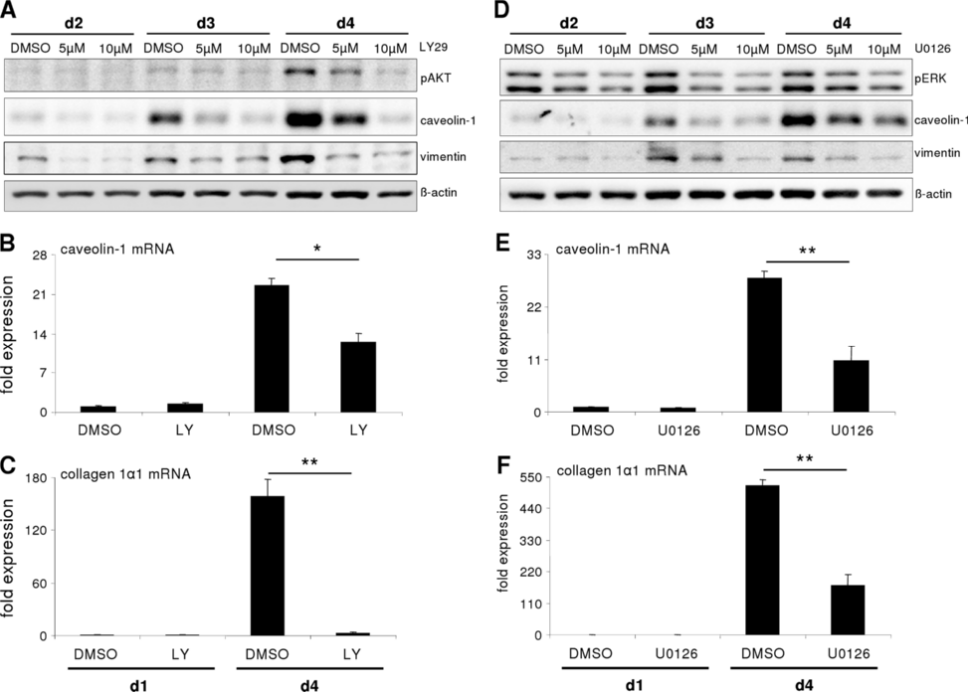
**The AKT and ERK pathways are required for caveolin-1 induction.** (**A**) Blocking AKT activation with Ly294002 decreased caveolin-1 upregulation on protein and (**B**) mRNA level (p = 0.018). (**C**) Induction of Collagen 1α1 mRNA, a mesenchymal marker, was completely abolished when AKT signaling was blocked (P < 0.001). (**D**) Inhibition of ERK activation via the small molecule inhibitor U0126 (U5: 5 μM; U10: 10 μM) culminated in strongly reduced caveolin-1 induction on protein and (**E**) mRNA level (p < 0.001). (**F**) ERK affects upregulation of Collagen 1α1 (p < 0.001) for mRNA expression studies, U0126 was used at a conc. of 10 μM.

Due to a recent report about the relevance of MAPK/ERK and AKT signaling pathways in modulating hepatocyte plasticity in monolayer culture, and the observation of affected ERK and AKT phosphorylation upon FAK/Src inhibition (Figure 2B and [3]), we intended to define the relevance of these pathways on caveolin-1 expression. Blocking culture dependent AKT activation with Ly294002 inhibitor sig nificantly reduced caveolin-1 protein and mRNA levels (Figure 4A, B). As also Collagen 1α1 induction was significantly blunted (Figure 4C), inhibition of AKT affected hepatocyte dedifferentiation and caveolin-1 induction. Next, U0126 was applied to interfere with ERK1/2 activity during hepatocyte culture on collagen monolayer. This led to reduced caveolin-1 and Collagen 1α1 expression, similar to the result obtained upon AKT pathway blunting (Figure 4D-F). In summary, both ERK1/2 and AKT pathways influenced hepatocyte dedifferentiation (vimentin, Figure 4A, D) and caveolin-1 expression.

### Snai1 is not involved in hepatocyte dedifferentiation and caveolin-1 induction

**Figure 5:**
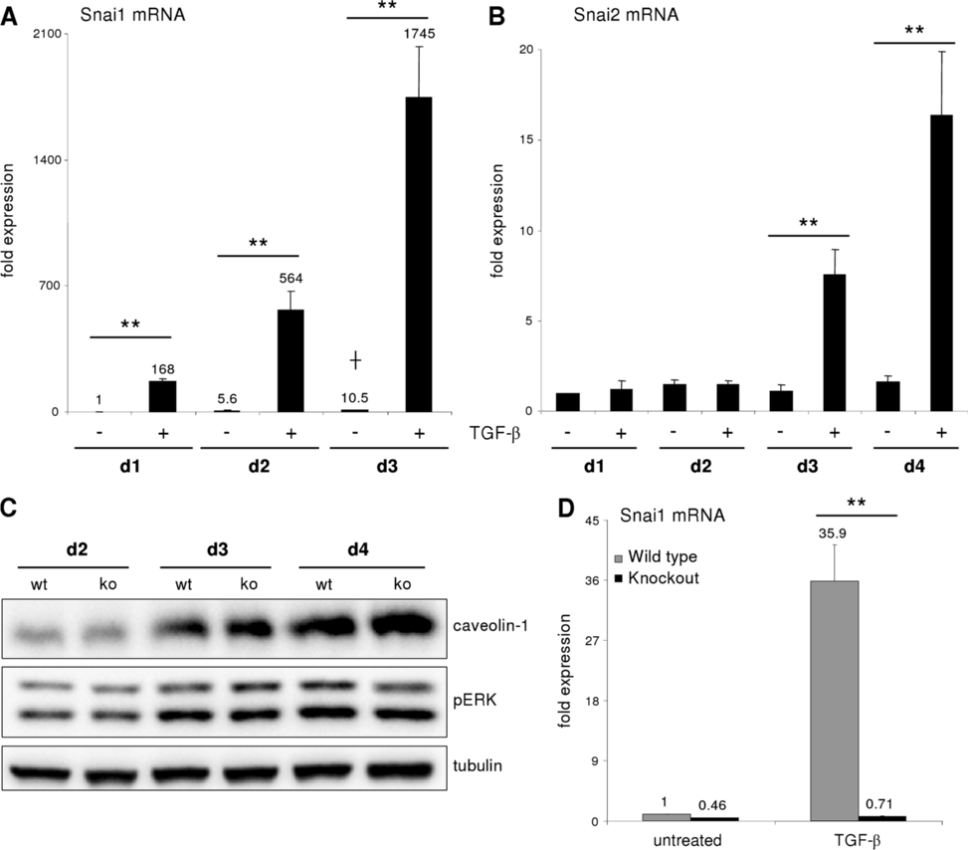
**The Snai1 family is not involved in intrinsic dedifferentiation and caveolin-1 upregulation.** (**A**) Snai1 is slightly upregulated during dedifferentiation and tremendously and time dependently induced by TGF-β1 stimulation (**p < 0.005 at all time points when comparing untreated with TGF-β1; p < 0.05 untreated d3 vs. untreated d1). (**B**) No change of Snai2 (Slug) expression could be detected in hepatocytes cultured for 4 days. Beginning on day 3, TGF-β did increase its expression (p ≤ 0.002 at d3 and d4 comparing expression of untreated vs. TGF-β1). (**C**) Dedifferentiating hepatocytes from wild type and Snai1 ko mice were analyzed for dedifferentiation events with Western blot. No alterations in ERK activation and caveolin-1 induction were observed. (**D**) Proof of hepatocyte specific Snai1 knockout. Basal Snai1 expression was very weak in wild type hepatocytes (based on qPCR data) and almost absent in Snai1 ko hepatocytes. Induction of Snai1 expression was triggered by TGF-β1 stimulation for 6 h. Control hepatocytes responded with strong induction, whereas Snai1 knockout hepatocytes did not induce expression (p < 0.001).

Considering the unique role of Snail transcription factor family in driving EMT, we tested whether Snai1 and Slug (Snai2) were involved in the culture dependent dedifferentiation process of hepatocytes. Intriguingly, during culture, Snai1 mRNA levels only slightly increased. As a potent mediator of EMT, TGF-β was previously shown to induce Snai1 expression. Accordingly, TGF-β treatment tremendously increased Snai1 expression in a time dependent manner (Figure 5A). Additionally, we evaluated a potential role of Slug during intrinsic dedifferentiation. We found Slug expression levels unchanged during 4 days of culture (Figure 5B) and thus rule out a potential function in the process of dedifferentiation. However, TGF-β1 induced a late and in comparison to Snai1, moderate Slug induction at day 3 and 4 of treatment (up to 16-fold after 4 day treatment). Therefore, Slug is not an early EMT mediator of TGF-β in hepatocytes.

To further confirm that Snai1 is not involved in the regulation of caveolin-1 expression, we showed that hepatocytes isolated from hepatocyte specific Snai1 knockout mice underwent culture dependent dedifferentiation and upregulated caveolin-1 expression comparable to controls (Figure 5C). The increase of pERK during dedifferentiation is also not affected by Snai1 expression, indicating independency of a Snai1 mediated mechanism. To prove the conditional Snai1 knockout, mRNA levels of Snai1 under basal conditions and after 6 h TGF-β treatment were investigated. Basal expression of Snai1 was weak in controls, and strongly reduced in hepatocytes from Snai1 ko mice. Upon TGF-β treatment, Snai1 expression boosted within 6 h in wild types up to 35 fold whereas Snai1 ko hepatocytes did not induce significant expression (Figure 5D). These observations suggested that culture induced hepatocyte dedifferentiation does not resemble a classical EMT due to Snai1 independency.

### TGF-β attenuates culture mediated caveolin-1 upregulation

**Figure 6:**
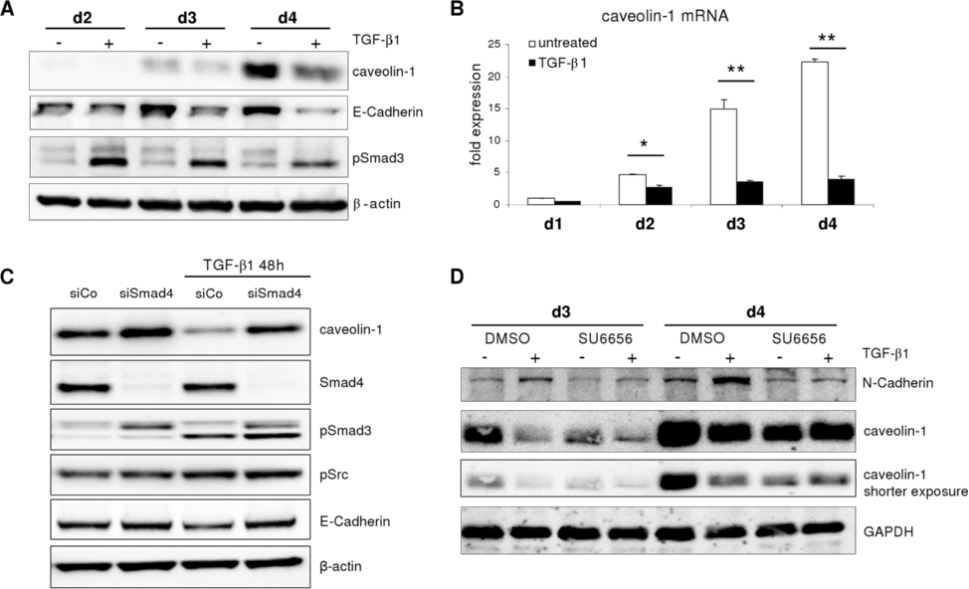
**Effects of TGF-β1 on caveolin-1 induction.** (**A**) TGF-β1 treated hepatocytes display attenuated caveolin-1 protein upregulation. TGF-β1 dependent EMT induction is reflected by E-Cadherin downregulation. (**B**) Similar to the protein regulation, mRNA levels of caveolin-1 are less induced compared to corresponding controls (p ≤ 0.001 after 2, 3 and 4 days). (**C**) Knockdown of Smad4 attenuates TGF-β1 mediated caveolin-1 repression. (**D**) Inhibition of Src using SU6656 (15 μM) blocked intrinsic and TGF-β mediated upregulation of N-Cadherin and caveolin-1.

As the above findings enabled a clear discrimination between culture induced dedifferentiation and TGF-β mediated EMT, it was of interest to determine whether TGF-β was able to induce caveolin-1 expression in primary hepatocytes. To test this hypothesis, monolayer cultured hepatocytes were treated with TGF-β for several days and subsequently caveolin-1 expression was analyzed. Caveolin-1 protein levels ascended over time in the controls, but to a lesser extent in cells treated with TGF-β (Figure 6A). This was paralleled by a weaker increase of caveolin-1 mRNA expression upon TGF-β stimulation during culture (Figure 6B). To determine whether the attenuation of caveolin-1 induction by TGF-β was Smad or non-Smad pathway dependent, Smad4 was knocked down to abrogate the canonical Smad pathway. TGF-β stimulation in Smad4 knock down hepatocytes did not lead to a reduction in caveolin-1 protein levels, as compared to controls (Figure 6C). Furthermore, Smad4 knockdown yielded in Smad3 hyperactivation, likely due to reduced nuclear shuttling with subsequent attenuated dephosphorylation and degradation of phospho-Smads. Noteworthy, Src phosphorylation was triggered by TGF-β stimulation, and this was not altered by knock down of Smad4.

As the relevance of Src in inducing caveolin-1 was evident, Src phosphorylation was blocked by SU6656 and cells were subsequently treated with TGF-β.As shown in Figure 6D, TGF-β reduced caveolin-1 expression in controls. SU6656 also affected caveolin-1 expression compared to untreated controls. When TGF-β and SU6656 were combined, no additive effect on expression was detectable. These findings argue for a dominant role of the Smad pathway on caveolin-1 repression, capable of overruling the Src axis. We therefore conclude that TGF-β is not able to induce caveolin-1 in normal epithelial hepatocytes.

### TGF-β induces caveolin-1 in low caveolin-1 expressing HCC cell lines

**Figure 7:**
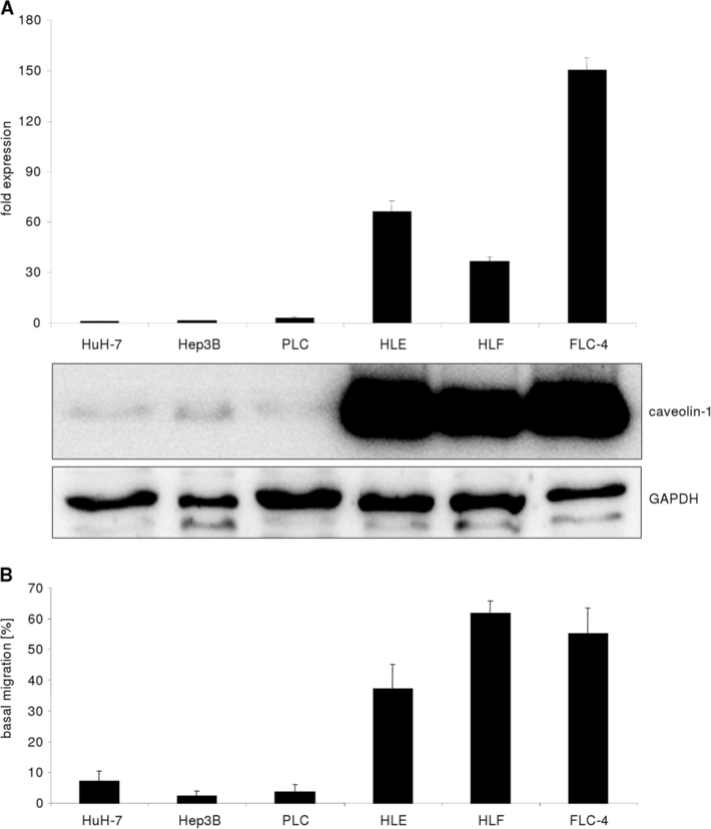
**HCC cell lines and caveolin-1 expression.** (**A**) HUH-7, Hep3B and PLC/PRF/5 are weak caveolin-1 expressing cells (both on mRNA and protein level, qPCR data normalized to 18S rRNA), whereas the dedifferentiated lines HLE, HLF and FLC-4 demonstrate high expression levels. (**B**) Based on elevated basal migration capacity, assessed with the transwell assay, HLE, HLF and FLC-4 are considered dedifferentiated (see text for details).

**Figure 8:**
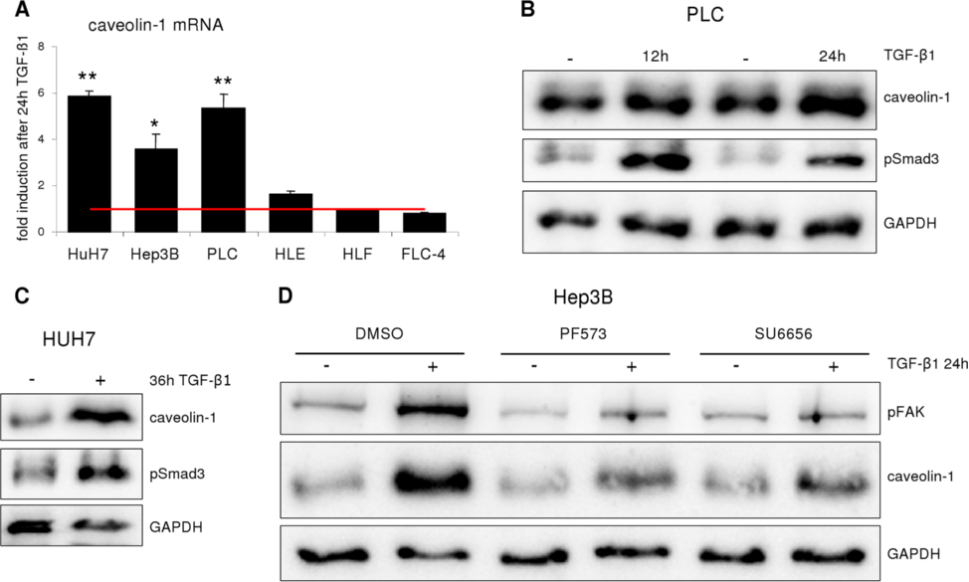
**TGF-β induces caveolin-1 expression in a subset of HCC cell lines.** (**A**) qReal-time analysis of TGF-β1 treated HCC cell lines. The low caveolin-1 expressing lines respond to TGF-β via an upregulation of expression (*p < 0.05; **p < 0.01), whereas HLE, HLF and FLC-4 do not change expression upon TGF-β1 challenge. (**B**) Western blot of PLC/PRF/5 lysates revealed caveolin-1 upregulation after 12 and 24 h of TGF-β1 treatment. (**C**) HUH-7 cells require 36 h TGF-β1 treatment to enhance caveolin-1 protein expression. (**D**) Hep3B cells strongly elevate caveolin-1 expression after 24 h of TGF-β1 treatment. Blocking FAK or Src phosphorylation by applying the chemical inhibitors PF573228 (4 μM) or SU6656 (15 μM) abolished the induction of caveolin-1 protein expression upon TGF-β1 stimulation.

Caveolin-1 has been linked to cancer, including HCC. Several studies correlated caveolin-1 expression and prognosis of the patient. Except one study, increased caveolin-1 levels have been linked to poor prognosis [9-12]. Six HCC cell lines, namely Hep3B, HUH-7, PLC/PRF/5, FLC-4, HLE and HLF, were screened for caveolin-1 expression and we found marked differences on both mRNA and protein level (Figure 7A). Previously, Hep3B, HUH-7 and PLC/PRF/5 were classified as differentiated and HLE and HLF as dedifferentiated HCC cell lines [16]. Noteworthy, FLC-4 cell line has an epithelial phenotype and was reported to demonstrate hepatocyte like functions [17]. However, these cells exhibit elevated basal migration capacity (Figure 7B) and have undergone the E- to N-Cadherin switch (not shown). Based on these observations, FLC-4 were assigned as “dedifferentiated” together with HLE and HLF cells. Considering the tumor promoting function of TGF-β in HCC, the cell lines were analyzed for TGF-β regulation of caveolin-1 expression. Interestingly, low expressing cell lines (Hep3B, HUH-7 and PLC/PRF/5) respond to TGF-β stimulation with significant upregulation of caveolin-1 expression (Figure 8A) on mRNA level. Similar results were obtained on protein level, although the kinetics of upregulation differed (Figure 8B-D). As the low expressing cell lines show a more differentiated phenotype, as compared to the high expressing ones, it can be hypothesized that TGF-β mediated cancer EMT is accompanied with an increase of caveolin-1 expression. Due to the implications of the FAK/Src axis on caveolin-1 expression [14], Hep3B were treated with PP2 or PF573228 to inhibit Src and FAK phosphorylation and subsequently stimulated with TGF-β1 for 24 h. Blocking of Src/FAK affected caveolin-1 induction (Figure 8D). To conclude, low caveolin-1 expressing HCC cell lines induce its expression upon TGF-β challenge via a FAK/Src-dependent pathway.

## Discussion

Hepatocyte dedifferentiation in collagen monolayer culture is a major obstacle for toxicity screening. During culture, hepatocyte metabolic functions are altered due to downregulation of metabolic enzymes including the broad family of Cyp enzymes [18,19]. Polarity establishment and maintenance are critical processes to regulate hepatocyte function and therefore in focus of research. Hepatocyte dedifferentiation impressively documents the cellular plasticity and evidences that the differentiation status *in vivo* does not have to be terminal. A recent intriguing finding underlining hepatocyte plasticity has been reported by Sahin and co-workers, who described differentiation of hepatocytes into liver progenitor cells [20]. Others made observations of EMT during hepatocellular cancer progression. Interestingly, primary hepatocytes have also been shown to undergo EMT upon TGF-β stimulation *in vitro*[7]. In contrast, *in vivo* EMT of hepatocytes during liver damage and fibrogenesis has recently been declined, although this was mainly related to transdifferentiation into myofibroblasts [21] and does not exclude phenotypical changes of hepatocytes into other directions.

*In vitro*, a distinction between intrinsic hepatocyte dedifferentiation and TGF-β mediated EMT has not yet been drawn. A recent study describes the capability of TGF-β to induce caveolin-1 expression in NMuMG and NT2/D1 cells lines, which has been linked to FAK/Src signaling [14]. Additionally, in a hepatocyte cell line (E14 MMH), TGF-β mediated EMT was shown to require FAK signaling [22]. Furthermore, intrinsic hepatocyte dedifferentiation in culture has also been connected to FAK/Src signaling [3]. Indeed, our study defines that FAK/Src activity is the driving force of hepatocyte dedifferentiation and caveolin-1 upregulation and thus, the FAK signaling pathway is implicated in TGF-β triggered effects. During intrinsic hepatocyte dedifferentiation, the downstream signaling routes MEK/ERK and PI3K/AKT are activated and subsequently regulate the induction of caveolin-1. Noteworthy, the dedifferentiation process in monolayer culture primes hepatocytes for proliferation as shown recently by microarray analysis and therefore may reflect a phenotype contributing to liver regeneration [23]. Due to linkage of caveolin-1 to proliferation in many settings and cell types [24], it might as well function in modulating hepatocyte proliferation. In sharp contrast, the EMT inducing TGF-β/Smad signaling pathway is overruling the above FAK/Src mediated signals and does not increase caveolin-1 levels in hepatocytes. In this context, the EMT promoting transcription factor Snai1 is induced weakly during culture and is strongly upregulated upon TGF-β treatment. This finding is consistent with the observation that the epithelial marker E-Cadherin is not downregulated on protein level during culture, although mesenchymal markers are induced. However, E-Cadherin localization at the plasma membrane is affected and therewith tight junction formation is compromised, leading to reduced cell-cell adhesion (but less compared to TGF-β mediated effects), a feature of mesenchymal cell types. TGF-β challenge, however, led to reduced E-Cadherin expression, which is mediated by Snai1 repression of the gene. For further delineation, upregulation of caveolin-1 and induction of mesenchymal markers are discrete from Snai1 function. Additionally, induction of mesenchymal markers and caveolin-1 are likely non-related events, as TGF-β is inducing the mesenchymal phenotype without increasing caveolin-1 expression. Further studies will shed light on the different mechanisms regulating different steps of the hepatocyte differentiation programmes.

Recognizing FAK/Src signaling as an important driver of caveolin-1 expression in hepatocytes, it is worth speculating about their microenvironment during disease development. During fibrogenesis and cancer development, the liver’s microarchitecture changes, comprising upregulation of extracellular matrix deposition, increased liver stiffness and a shift to fibril forming collagens (mainly type I and III). Integrins are sensors of the extracellular milieu and subsequently signal status information into the cell, commonly involving FAK/Src. Thus, the extracellular matrix composition of the liver may be of relevance for changes in caveolin-1 expression and subsequent modulation of hepatocyte function also *in vivo*.

Hepatocellular cancer commonly develops after decades of liver fibrosis/cirrhosis, where extensive matrix remodelling has taken place. Therefore, the increase of caveolin-1 in progressed HCC likely results from matrix signals (but other regulatory factors, e.g. cholesterol, have been described [25,26]). However, we also found that TGF-β is capable to increase caveolin-1 expression in some HCC cells lines. A common feature of this observation was the relatively low basal expression of caveolin-1 as in contrast, the dedifferentiated, high caveolin-1 expressing cell lines did not increase expression upon TGF-β stimulation. This points to an interesting aspect. TGF-β is considered as a tumor suppressor; however, frequently and especially in progressed disease stages, its function may switch to a tumor promoter [27]. In our study, we define caveolin-1 as a non-target of TGF-β in untransformed hepatocytes, whereas in early transformed (differentiated HCC) cancer cell lines, TGF-β is mediating enhanced expression of caveolin-1 and therewith may promote tumor proliferation and migration/invasion, functions that have been attributed to caveolin-1. With regard to EMT, in hepatocytes, caveolin-1 cannot be considered as an indicator (or marker) of EMT processes as it does not occur as a TGF-β target gene during this process. However the role in cancer EMT has yet to be defined.

## Conclusion

Morphological similar processes of intrinsic and TGF-β induced hepatocyte dedifferentiation underlie distinct molecular mechanisms including activation of signalling pathways and induction of target genes. Snai1 is a mediator of TGF-β triggered EMT, whereas it is not involved in intrinsic differentiation. Furthermore, caveolin-1, suggested as an EMT marker, is repressed by TGF-β and strongly induced during hepatocyte culture. In contrast, caveolin-1 turns into a TGF-β target in transformed hepatocytes (HCC cell lines), where Smad and non-Smad/FAK/Src signalling pathways contribute. These findings imply that a further delineation of programmes leading to hepatocyte plasticity, depending on culture conditions, is inevitable.

Additionally, the TGF-β dependent transcriptional patterns are strongly depending on the differentiation status of the hepatocyte. Further research on primary hepatocytes and cell lines will generate insights into the integration of Smad and non-Smad pathways leading to a unique cell response, especially in context of TGF-β’s functional switch from a tumor suppressor towards a tumor promoter in cancerogenesis.

## Materials and methods

### Cell culture

Primary hepatocytes were isolated from livers of male C57BL/6 wild type mice by collagenase perfusion. Hepatocytes then were plated on collagen coated plates and cultured in Williams E medium supplemented with Pen/Strep, L-glutamine, dexamethasone and FCS [6]. After attachment (4 h), cells were cultured in medium lacking FCS. For analyzing the function of Snai1 during intrinsic dedifferentiation, hepatocytes were isolated from B6;129-TgN(Alb-Cre^-/+^)Snai1^tm1MhM^ mice with a hepatocyte specific deletion of functional Snai1 gene [28]. From these animals and corresponding wild type mice, hepatocytes were isolated as described above. HCC cell lines used in this study: HUH-7, Hep3B, PLC/PRF/5, HLE, HLF, and FLC-4. For stimulation experiments, FCS free medium containing inhibitors and/or TGF-β was refreshed every 24 h.

### Reagents and antibodies

Recombinant TGF-β1 was purchased from Peprotech (Hamburg, Germany) and used at a final concentration of 5 ng/ml. Antibodies used: pAKT, pSrc527 (Cell Signaling), pSmad3 (epitomics), caveolin-1, GAPDH, pERK, pFAK397 and horseradish peroxidase-linked anti mouse and rabbit secondary antibodies were from SantaCruz. E-Cadherin (BD Biosystems), β-actin (Sigma-Aldrich), N-Cadherin, tubulin and vimentin were obtained from abcam. Inhibitors used in this study: PF573228 (Sigma-Aldrich), SU6656 (Calbiochem), PP2, LY294002, U0126 (Tocris).

### Immunofluorescence of F-actin

F-actin fibres were visualized using Alexa Fluor 488 phalloidin (invitrogen) and nuclei using Draq5 (alexis biochemicals). Cells were cultured on collagen coated glass slides and fixed with 2% PFA, permeabilized with 0.3% Triton-X 100 in PBS followed by a final 4% PFA fixation step. Fixed cells were incubated with phalloidin and Draq5 in a 1% BSA/PBS solution for 1 h. After washing with PBS, cells were mounted on object slides and images acquired using the confocal microscope Leica TCS SP2.

### Western blot analysis

Hepatocyte lysates were taken with RIPA buffer (50 mMTris-HCl (pH 7.5), 150 mMNaCl, 1% Nonidet P-40 (NP40), 0.5% sodium deoxycholate, 0.1% SDS, Proteases Inhibitor Cocktail, Phosphatase Inhibitor Cocktail II (Roche, Germany)). Protein concentration was determined using the DC protein assay (Bio-Rad). 30 μg protein per sample were separated by SDS-PAGE and blotting was processed as described [29]. Then, membranes were developed using ECL solution and the Chemi-Smart (Vilber Lourmat) system.

### RNA isolation, reverse transcription and qRT-PCR

Total RNA was extracted using the RNAeasy Mini Kit (Qiagen). Subsequently, cDNA was synthesized from 1 μg RNA with the Quantitect Reverse Transcription kit (Qiagen). qRT-PCR was performed using taqman technology on a Stratagene Mx3005P. Snai1, Snai2, PPIA, caveolin-1 and Collagen 1α1 primer mixes were purchased from Applied Biosystems. For normalization of cell line data, 18S rRNA specific taqman primers were used.

### RNA interference

Transfection with siRNA was essentially performed as in [29]. In brief, RNAiMAX (invitrogen) was used as transfection reagent. A final concentration of 10 nM was chosen. Oligos were pools obtained from Dharmacon. Transfection was carried out 4 h after seeding of primary hepatocytes and transfection mix was incubated o/n. Stimulation experiments were conducted 36 h post transfection.

### Migration assay

For elucidating migration capacity of the different HCC cell lines, a transwell assay was applied. Transwell inserts (BD) were used (pore size 8 μm). 25,000 starved cells per insert were seeded. The upper compartment contained 1% BSA, whereas the lower part contained 10% FCS to induce migration. Cells were allowed to migrate for 13 h. Subsequently, cells from both compartments were trypsinized and an ATP assay (CellTiter-Glo, Promega) was conducted to evaluate % of migrated cells (values lower compartment divided by sum of both compartments).

### Densitometry

For quantitative analysis of Western blots, Aida Image Analyzer v. 4.25 was used. Each value was calculated from three independent experiments. Expression was normalized to GAPDH protein levels. Normalized control was set to 1 at each time point (day3 and day4).

### Statistics

Significance was calculated with the two-tailed Student’s t-test. Values were pooled from at least three independent experiments or as indicated.

## Abbreviations

EMT: Epithelial mesenchymal transition;HCC: Hepatocellular carcinoma;TGF-β: Transforming growth factor beta

## Competing interests

The authors declare that they have no competing interests.

## Authors’ contributions

CM designed, performed and analyzed experiments, wrote the manuscript, JD performed experiments with HCC cell lines; YL performed experiments and analysed data, wrote manuscript. FS did experiments with primary hepatocytes and analysed RNA and protein samples; SM designed experiments and helped drafting the manuscript, AM isolated primary hepatocytes and helped to draft manuscript, CC helped to draft the manuscript, SD supervised the experiments, analysed data and wrote the manuscript. All authors read and approved the final manuscript.

## References

[B1] Guillouzo A Guguen-Guillouzo C Evolving concepts in liver tissue modeling and implications for in vitro toxicology Expert Opin Drug Metab Toxicol 2008 4 1279 1294 10.1517/17425255.4.10.1279 18798698

[B2] Kim Y Rajagopalan P 3D hepatic cultures simultaneously maintain primary hepatocyte and liver sinusoidal endothelial cell phenotypes PLoS One 2011 5 e15456 21103392 10.1371/journal.pone.0015456 PMC2980491

[B3] Godoy P Hengstler JG Ilkavets I Meyer C Bachmann A Muller A Tuschl G Mueller SO Dooley S Extracellular matrix modulates sensitivity of hepatocytes to fibroblastoid dedifferentiation and transforming growth factor beta-induced apoptosis Hepatology 2009 49 2031 2043 10.1002/hep.22880 19274752

[B4] Baker TK Carfagna MA Gao H Dow ER Li Q Searfoss GH Ryan TP Temporal gene expression analysis of monolayer cultured rat hepatocytes Chem Res Toxicol 2001 14 1218 1231 10.1021/tx015518a 11559036

[B5] Pohlers D Brenmoehl J Loffler I Muller CK Leipner C Schultze-Mosgau S Stallmach A Kinne RW Wolf G TGF-beta and fibrosis in different organs - molecular pathway imprints Biochim Biophys Acta 2009 1792 746 756 10.1016/j.bbadis.2009.06.004 19539753

[B6] Weng HL Ciuclan L Liu Y Hamzavi J Godoy P Gaitantzi H Kanzler S Heuchel R Ueberham U Gebhardt R Profibrogenic transforming growth factor-beta/activin receptor-like kinase 5 signaling via connective tissue growth factor expression in hepatocytes Hepatology 2007 46 1257 1270 10.1002/hep.21806 17657819

[B7] Zeisberg M Yang C Martino M Duncan MB Rieder F Tanjore H Kalluri R Fibroblasts derive from hepatocytes in liver fibrosis via epithelial to mesenchymal transition J Biol Chem 2007 282 23337 23347 10.1074/jbc.M700194200 17562716

[B8] Parton RG Simons K The multiple faces of caveolae Nat Rev Mol Cell Biol 2007 8 185 194 17318224 10.1038/nrm2122

[B9] Cokakli M Erdal E Nart D Yilmaz F Sagol O Kilic M Karademir S Atabey N Differential expression of Caveolin-1 in hepatocellular carcinoma: correlation with differentiation state, motility and invasion BMC Cancer 2009 9 65 10.1186/1471-2407-9-65 19239691 PMC2656543

[B10] Tang Y Zeng X He F Liao Y Qian N Toi M Caveolin-1 is related to invasion, survival, and poor prognosis in hepatocellular cancer Med Oncol 2012 29 2 977 984 10.1007/s12032-011-9900-5 21416157

[B11] Yang SF Yang JY Huang CH Wang SN Lu CP Tsai CJ Chai CY Yeh YT Increased caveolin-1 expression associated with prolonged overall survival rate in hepatocellular carcinoma Pathology 2010 42 438 445 10.3109/00313025.2010.494293 20632820

[B12] Zhang ZB Cai L Zheng SG Xiong Y Dong JH Overexpression of caveolin-1 in hepatocellular carcinoma with metastasis and worse prognosis: correlation with vascular endothelial growth factor, microvessel density and unpaired artery Pathol Oncol Res 2009 15 495 502 10.1007/s12253-008-9144-7 19434519

[B13] Bosch M Mari M Herms A Fernandez A Fajardo A Kassan A Giralt A Colell A Balgoma D Barbero E Caveolin-1 deficiency causes cholesterol-dependent mitochondrial dysfunction and apoptotic susceptibility Curr Biol 2011 21 681 686 10.1016/j.cub.2011.03.030 21497090 PMC3409647

[B14] Bailey KM Liu J Caveolin-1 up-regulation during epithelial to mesenchymal transition is mediated by focal adhesion kinase J Biol Chem 2008 283 13714 13724 10.1074/jbc.M709329200 18332144 PMC2376249

[B15] Chen T George JA Taylor CC Src tyrosine kinase as a chemotherapeutic target: is there a clinical case? Anticancer Drugs 2006 17 123 131 10.1097/00001813-200602000-00002 16428929

[B16] Coulouarn C Factor VM Thorgeirsson SS Transforming growth factor-beta gene expression signature in mouse hepatocytes predicts clinical outcome in human cancer Hepatology 2008 47 2059 2067 10.1002/hep.22283 18506891 PMC2762280

[B17] Laurent T Murase D Tsukioka S Matsuura T Nagamori S Oda H A novel human hepatoma cell line, FLC-4, exhibits highly enhanced liver differentiation functions through the three-dimensional cell shape J Cell Physiol 2012 227 2898 2906 10.1002/jcp.23033 21960466

[B18] Kidambi S Yarmush RS Novik E Chao P Yarmush ML Nahmias Y Oxygen-mediated enhancement of primary hepatocyte metabolism, functional polarization, gene expression, and drug clearance Proc Natl Acad Sci USA 2009 106 15714 15719 10.1073/pnas.0906820106 19720996 PMC2747185

[B19] Yarmush ML Toner M Dunn JC Rotem A Hubel A Tompkins RG Hepatic tissue engineering. Development of critical technologies Ann N Y Acad Sci 1992 665 238 252 10.1111/j.1749-6632.1992.tb42588.x 1416606

[B20] Chen Y Wong PP Sjeklocha L Steer CJ Sahin MB Mature hepatocytes exhibit unexpected plasticity by direct dedifferentiation into liver progenitor cells in culture Hepatology 2012 55 2 563 574 10.1002/hep.24712 21953633 PMC3268884

[B21] Taura K Miura K Iwaisako K Osterreicher CH Kodama Y Penz-Osterreicher M Brenner DA Hepatocytes do not undergo epithelial-mesenchymal transition in liver fibrosis in mice Hepatology 2010 51 1027 1036 10.1002/hep.23368 20052656 PMC2906231

[B22] Cicchini C Laudadio I Citarella F Corazzari M Steindler C Conigliaro A Fantoni A Amicone L Tripodi M TGFbeta-induced EMT requires focal adhesion kinase (FAK) signaling Exp Cell Res 2008 314 143 152 10.1016/j.yexcr.2007.09.005 17949712

[B23] Zellmer S Schmidt-Heck W Godoy P Weng H Meyer C Lehmann T Sparna T Schormann W Hammad S Kreutz C Transcription factors ETF, E2F, and SP-1 are involved in cytokine-independent proliferation of murine hepatocytes Hepatology 2010 52 2127 2136 10.1002/hep.23930 20979052

[B24] Boscher C Nabi IR Caveolin-1: role in cell signaling Adv Exp Med Biol 2012 729 29 50 10.1007/978-1-4614-1222-9_3 22411312

[B25] Bist A Fielding PE Fielding CJ Two sterol regulatory element-like sequences mediate up-regulation of caveolin gene transcription in response to low density lipoprotein free cholesterol Proc Natl Acad Sci USA 1997 94 10693 10698 10.1073/pnas.94.20.10693 9380697 PMC23450

[B26] Dasari A Bartholomew JN Volonte D Galbiati F Oxidative stress induces premature senescence by stimulating caveolin-1 gene transcription through p38 mitogen-activated protein kinase/Sp1-mediated activation of two GC-rich promoter elements Cancer Res 2006 66 10805 10814 10.1158/0008-5472.CAN-06-1236 17108117 PMC4288740

[B27] Dooley S Weng H Mertens PR Hypotheses on the role of transforming growth factor-beta in the onset and progression of hepatocellular carcinoma Dig Dis 2009 27 93 101 10.1159/000218340 19546546

[B28] Murray SA Carver EA Gridley T Generation of a Snail1 (Snai1) conditional null allele Genesis 2006 44 7 11 10.1002/gene.20178 16397867

[B29] Meyer C Godoy P Bachmann A Liu Y Barzan D Ilkavets I Maier P Herskind C Hengstler JG Dooley S Distinct role of endocytosis for Smad and non-Smad TGF-beta signaling regulation in hepatocytes J Hepatol 2011 55 369 378 10.1016/j.jhep.2010.11.027 21184784

